# Key driving forces of desertification in the Mu Us Desert, China

**DOI:** 10.1038/s41598-017-04363-8

**Published:** 2017-06-21

**Authors:** Xunming Wang, Hong Cheng, Hui Li, Junpeng Lou, Ting Hua, Wenbin Liu, Linlin Jiao, Wenyong Ma, Danfeng Li, Bingqi Zhu

**Affiliations:** 1 Key Laboratory of Water Cycle & Related Land Surface Processes, Institute of Geographic Sciences and Natural Resources Research, Chinese Academy of Sciences, Beijing, 100101 China; 2 University of Chinese Academy of Sciences, Beijing, 100049 China; 3 State Key Laboratory of Earth Surface Processes and Resource Ecology, Beijing Normal University, Beijing, 100875 China; 4 Key Laboratory of Desert and Desertification, Cold and Arid Regions Environmental and Engineering Research Institute, Chinese Academy of Sciences, Lanzhou, 730000 China

## Abstract

The temporal trends and key driving forces of desertification in the Mu Us Desert are representatives of most arid regions of Asia with a high risk of desertification. We analyzed the significance of Aeolian transport on desertification in the Mu Us Desert by field investigations, sampling, wind tunnel experiments, particle size and nutrient measurements, and statistics on aeolian transport potentials. The results showed that high intensities of aeolian processes may result in low differences in aeolian transport despite differences in the underlying sediments. When high desertification occurred in the 1970s, the annual losses of the ammonium N, nitrate N, available K, and available P were approximately 116, 312, 46,436, and 1,251 kg km^−2^, respectively. After 2010, the losses were only 8, 20, 3,208, and 84 kg km^−2^, which were generally only 6.7% of those in the 1970s. The results showed that although human activity may trigger desertification, the dramatic decline of aeolian transport and low nutrient loss may be the key driving forces for the occurrence of rehabilitation in this region.

## Introduction

Arid Asia stretches from Northeast Asia to Central and West Asia and covers an area of 1.5 × 10^8^ km^2^, of which more than 70% is covered by sand dunes, sand sheets, gravel surfaces, and steppes. In addition, the annual mean precipitation is less than 500 mm, and the aridity index is less than 0.50^1^ (Figure S1). These areas are usually managed as traditional pastoral and agricultural systems, and desertification occurrence could seriously endanger and jeopardize the existence of approximately 350 million people^2^. Among the regions with high risks of desertification in arid Asia, the Mu Us Desert (S1) is a representative area where human activity is usually considered to be a key driving force of desertification^3, 4^. The major forms of desertification include arable land and grassland degradation, anchored or semi-anchored dune reactivation^5^, and under the background of global warming the expansions of drylands and of the erosion-induced land degradation^6^. With the occurrence of desertification, the nutrients in soil such as nitrogen (N), phosphorus (P), and potassium (K) are eroded^7, 8^, and the soil fertility decreases^9, 10^, which consequently affects the regional ecosystems^11–14^.

**Figure 1:**
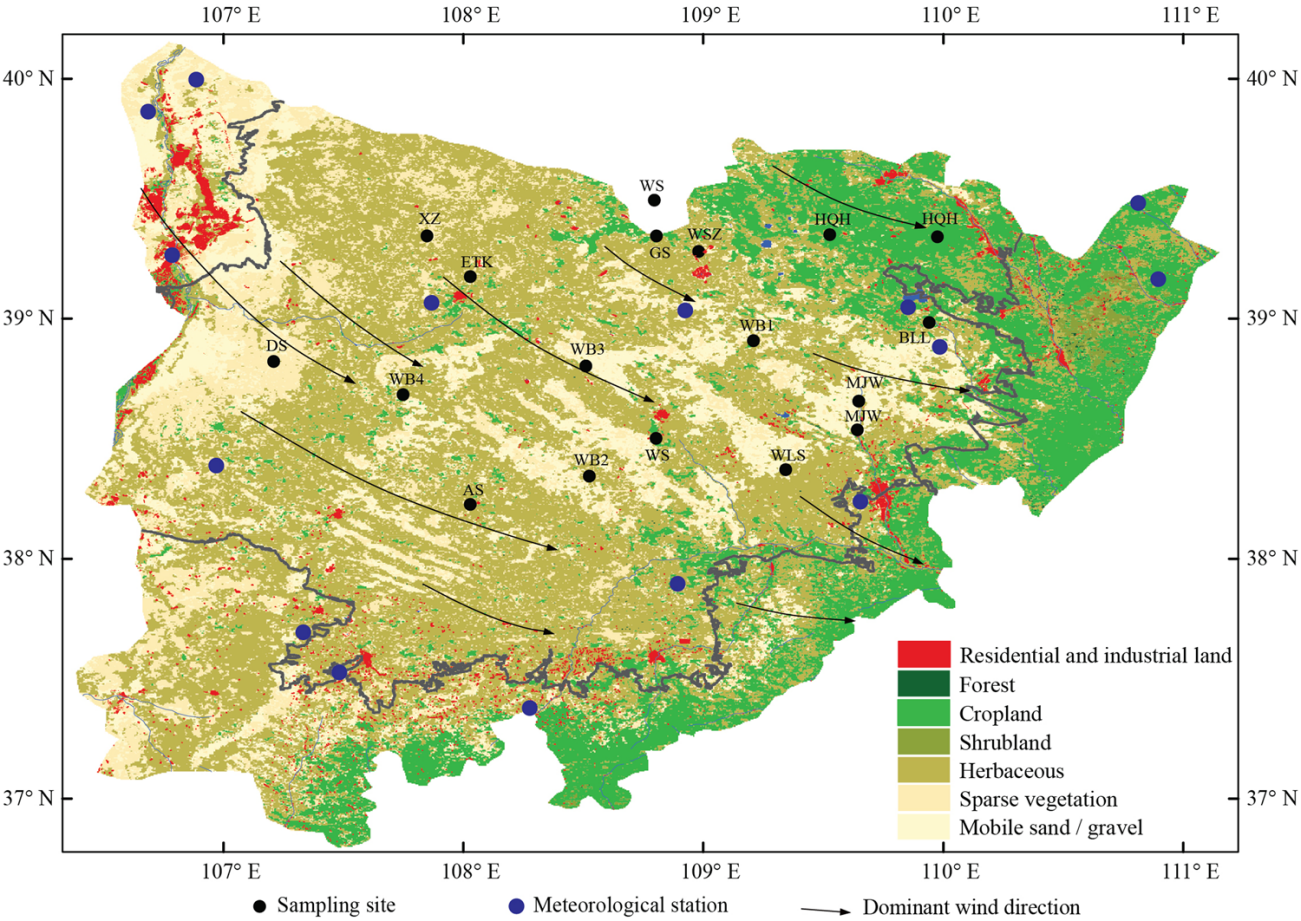
Locations of the Mu Us Desert, sample sites and land uses. The areas outlined in grey indicate areas affected by desertification from the mid-1970s to 2010. The black and blue dots indicate the locations of the sampling sites and meteorological stations, respectively, used in this study (The figure was finished using Arcgis software (version 10.1, ESRI Inc., Redlands, California, USA), which can be downloaded from the internal network of Institute of Geographic Sciences and Natural Resources Research, Chinese Academy of Sciences.).

Despite some spatial differences in desertification trends over the past decades, high desertification occurred in the 1970s, whereas from the early 2000s to the present rehabilitation occurred in most regions of arid Asia, especially in China^15, 16^. Some have argued that desertification is triggered by human activities, whereas others have insisted that climate change may be the key factor influencing rehabilitation^17–19^. The key driving forces of desertification in arid Asia, however, are still poorly understood. We analyzed the driving forces of desertification in the Mu Us Desert (Fig. 1 and S1) over the past several decades by collecting filed samples (S2), employing wind tunnel experiments (S3), and conducting statistical analysis on aeolian transport potentials (S4–S5). The results demonstrated that aeolian transport occurrence force, rather than human activity, was the key driver of desertification in the Mu Us Desert over the past several decades.

## Results and Discussion

### Variations in particle sizes of surface soils under aeolian processes

**Figure 2:**
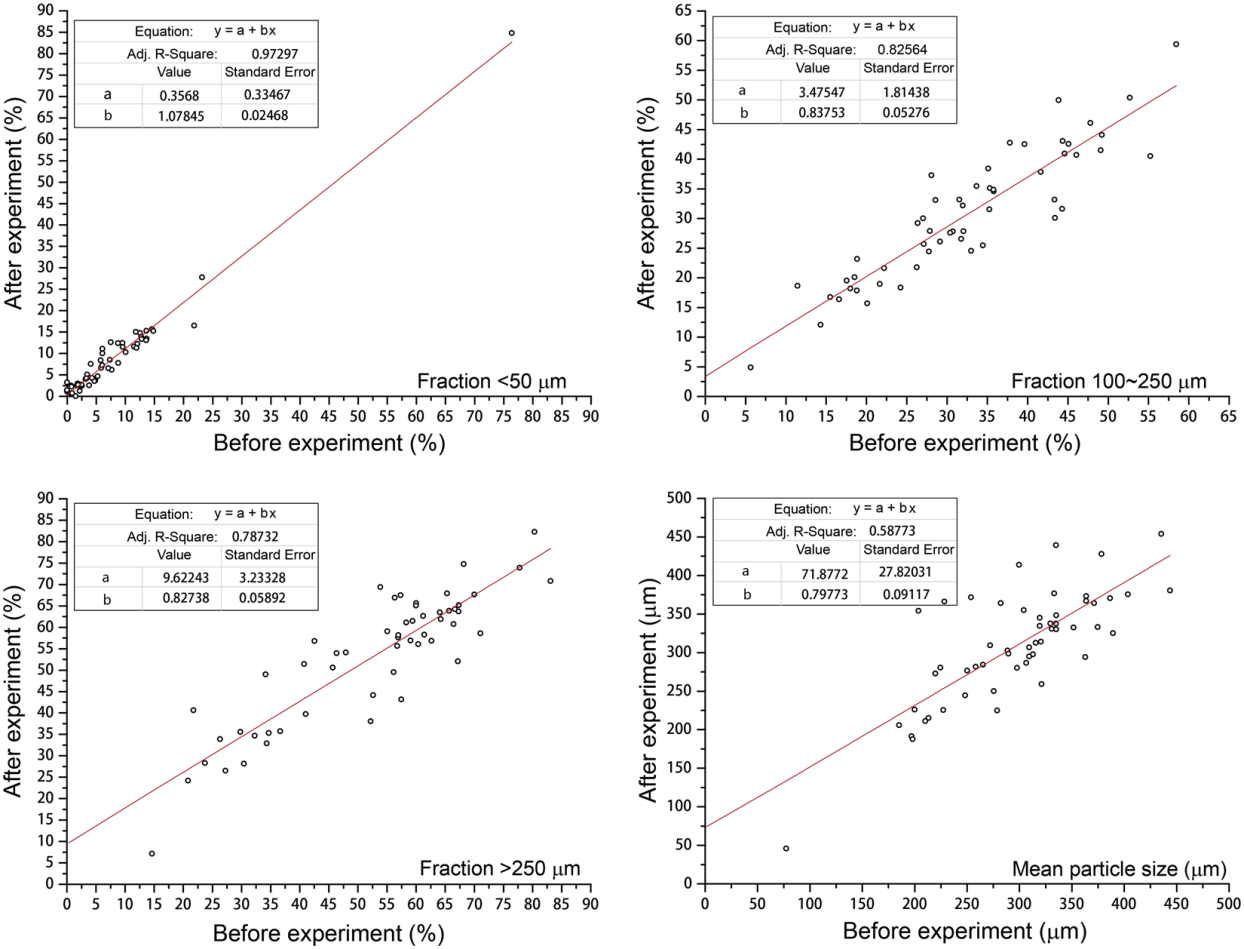
Scatterplots for the relationship of particle size fractions of surface soil before and after wind tunnel experiments.

Aeolian processes result in great variations in the components of surface soils. Before and after the wind tunnel experiments, little difference in the contents of the fine fraction (<50 µm in diameter) was observed. However, relatively coarser fractions were left after the experiments (Fig. 2). Contents of fractions with diameters ranging between 100 and 250 µm and of >250 µm in surface soils were higher by approximately 2.5% and 10% after aeolian processes, respectively. The results show that aeolian processes may coarsen the surface soils, leading to nutrient loss, and may decrease the water-holding capacity of surface soils.

### Temporal variations in aeolian transport

**Figure 3:**
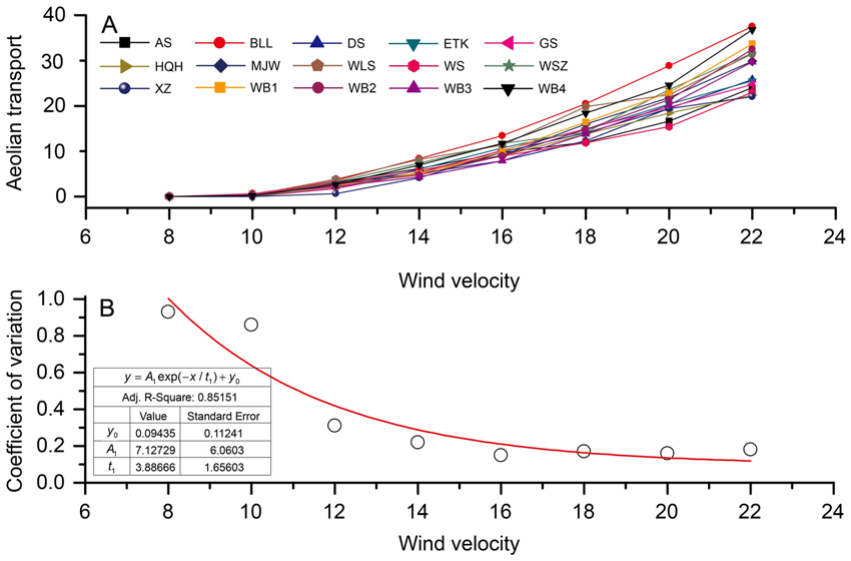
Average aeolian transport (g m^2^ s^−1^) (**A**) and the coefficient of variation (*CV*, **B**) in wind tunnel experiments under different wind velocities (m s^−1^). The *CV* is expressed as $CV=\frac{SD}{Mean}$, where *SD* and *Mean* refer to the standard deviation and average, respectively.

The average aeolian transport of 75 samples collected in 15 sites is shown in Fig. 3A. The results showed that the average aeolian transports varied from 0.01 to 28.71 g m^−2^ s^−1^, with a coefficient of variation (*CV*) of 0.37, under wind velocities ranging from 8 to 22 m s^−1^. The *CV* in aeolian transport among the samples decreased with the increase in wind velocity (Fig. 3B). This result indicates that the effects of variation in land use, degradation degrees, and soil properties on aeolian transport under high wind velocities are less than that under low wind velocities. High intensities of aeolian transport may have similar effects on desertification, despite the differences in spatial and temporal variations of the components of the underlying soils in the region.

**Figure 4:**
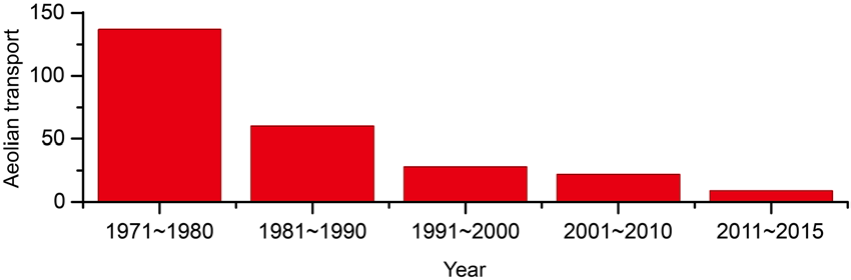
Temporal variation in aeolian transport (10^4^ ton km^−2^) in the Mu Us Desert.

With the occurrence of aeolian processes, nutrients are lost, mobile dunes and sand sheets develop on the surface, the soil fertility and biomass decrease, and desertification occurs. Wind tunnel experiments and statistical results showed that there were also obvious temporal variations for aeolian transport in the Mu Us Desert (S4). For example, in the 1970s the intensity of aeolian transport was approximately 137 × 10^4^ T km^−2^, whereas the value was only 9 × 10^4^ ton km^−2^ from 2011 to the present, representing only 6.6% of that in the 1970s (Fig. 4). The dramatic decline of aeolian transport from 2011 to the present showed that there was little development of mobile dunes and sand sheets, which consequently benefited rehabilitation in this region.

### Nutrient loss intensities under different wind velocities

**Figure 5:**
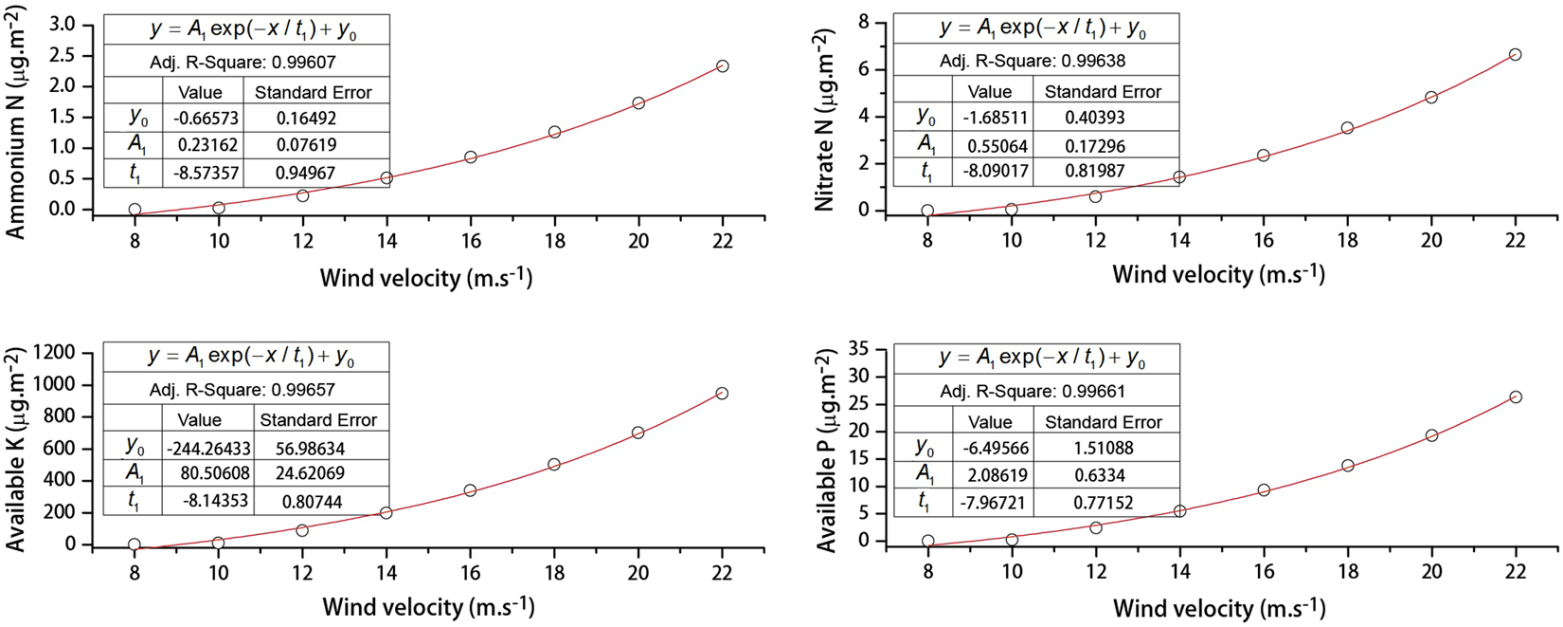
Nutrient loss under different wind velocities.

The average contents of ammonium N, nitrate N, available K and available P in the surface soils were 0.08 (±0.05, SD), 0.25 (±0.52), 35.18 (±20.26), and 0.95 (±0.46) mg kg^−1^, respectively. There were no significant correlations between the nutrient contents and contents of different particle size fractions, except for the 50 ~ 100 µm and 200 ~ 250 µm fractions that correlated with the contents of available K and available P, respectively (S4). Under a wind velocity of 8 ~ 22 m s^−1^, the nutrient loss increased with increasing wind velocity (Fig. 5). For example, under a wind velocity of 8 m s^−1^, the contents of ammonium N, nitrate N, available K and available P were only 0.0004, 0.0010, 0.2043, and 0.0053 µg m^−2^, respectively. Under a wind velocity of 22 m s^−1^, the values were 2.3258, 6.6414, 947.0200, and 26.3171 µg m^−2^, respectively. These results suggest that high intensities of aeolian processes may increase nutrient loss and enhance desertification in the region.

### Nutrient loss and its importance on desertification

**Figure 6:**
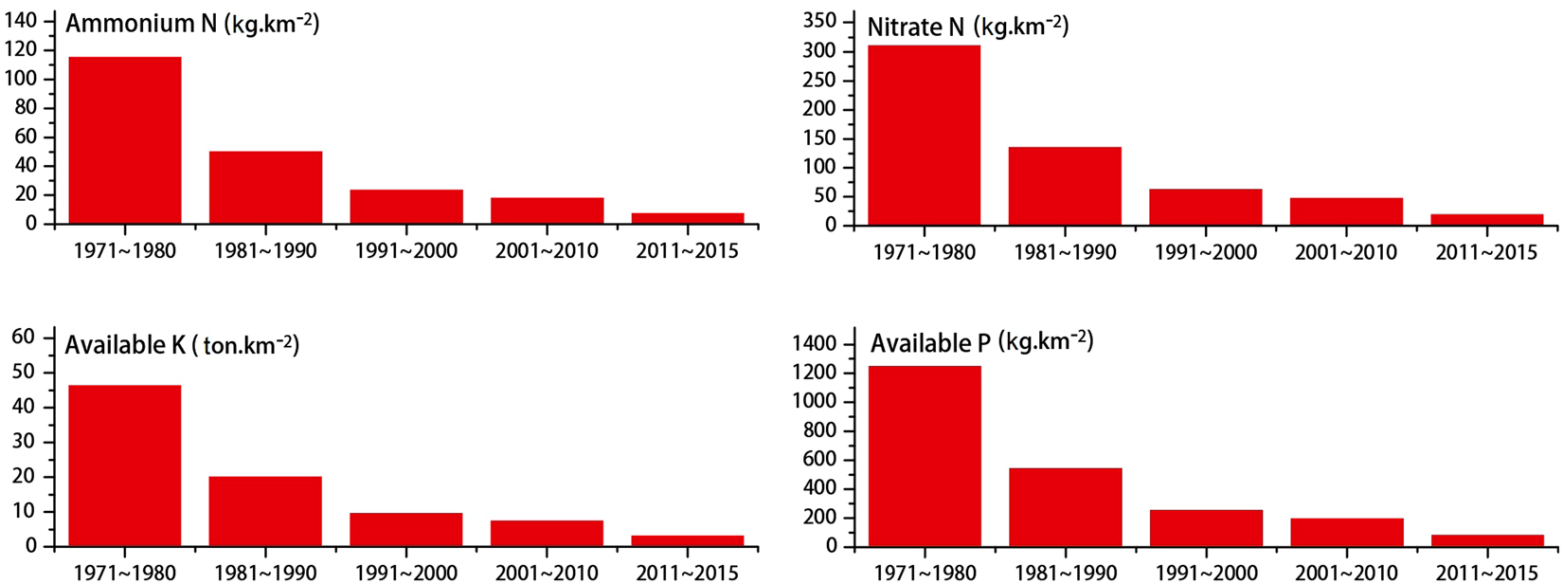
Nutrient loss under different wind velocities in the Mu Us Desert during different periods.

Based on the combined results of wind tunnel experiments, nutrient content analyses, and the statistics of sand-driving winds (S4), there were obvious temporal variations in the nutrient loss that may have played an important role in the desertification or rehabilitation in the region over the past several decades (Fig. 6). The dramatic decline of nutrient loss was mainly because of the significant decrease of aeolian transport potential in the region. For example, in the 1970s the losses of ammonium N, nitrate N, available K and available P were 116, 312, 46,436, and 1,251 kg km^−2^, respectively, whereas from 2011 to the present the losses were only 8, 20, 3,208, and 84 kg km^−2^, respectively. Nutrient loss of the surface soils from 2011 to the present was generally 6.8% of that in the 1970s.

Some researchers^20^ have suggested that human activities such as grazing, reclamation, and deforestation were the key forces driving desertification in the Mu Us Desert. However, over the past several decades there were several fluctuations of desertification in the region. For example, monitoring showed that desertification mainly occurred in the 1970s and 1980s, whereas rehabilitation occurred in most areas of the Mu Us Desert from 1990s to the present ^15, 16, 21, 22^. In the mid-1970s, 2000, 2005 and 2010, the areas of mobile dunes and sand sheets in China triggered by desertification were approximately 95,000, 107,000, 117,000, 95,000, and 82,000 km^2^, respectively^23^. From 2010 to 2015, the areas of mobile dunes and sand sheets continuously decreased throughout China^15, 16^. Although human activities continuously increased in areas with high risks of desertification in China over the past several decades^5, 18^, rehabilitation occurred in these regions. Additionally, drought may be one of the key controllers of desertification in some regions^24^. From 1960 to 2009, annual precipitation decreased at a rate of 11.383 mm 10a^−1^ in the Mu Us Desert^25^, and the variation in precipitation may have contributed to desertification. The decrease in aeolian transport was therefore the key driving force for the occurrence of rehabilitation, despite the importance of human activities on desertification in the region.

## Conclusions

Under high intensities of aeolian processes, there were no obvious differences in aeolian transports despite some variations in the components of the underlying soils. Although human activities played important roles, the temporal trends in desertification showed that the dramatic decline of aeolian transport potentials was the key driving forces for the occurrence of rehabilitation in the Mu Us Desert from 2011 to the present. Desertification in the 1970s led to the loss of ammonium N, nitrate N, available K, and available P at rates of approximately 116, 312, 46,436, and 1,251 kg km^−2^. From 2010 to the present, the losses were 8, 20, 3,208, and 84 kg km^−2^, respectively, representing only 6.7% of the losses of the 1970s. The results showed that although human activities played important roles in desertification, the distinct decrease of aeolian transport and nutrient loss may be the key driving forces for the occurrence of rehabilitation in the Mu Us desert.

## Material and Methods

The selected area for desertification driving force analyses was the Mu Us Desert in Central China (Fig. 1 and S1), which has been identified as a region of intense desertification^1^. The dominant soil type is aeolian sand, and major landscapes include anchored, semi-anchored and mobile dunes, and arable lands. Dominant natural vegetation in this region includes *Salix psammophila* C. Wang et Chang Y. Yang, *Caragana microphylla* Lam., *Stipa grandis* P. Smirn.*, Stipa bungeana* Trin.*, Agropyron cristatum* (L.) Gaertn.*, Thymus mongolicus* Ronniger, *Caragana tibetica* Kom.*, Oxytropis aciphylla* Ledeb.*, Nitraria sibirica* Pall. and *Kalidium foliatum* (Pall.) Moq., with most species being annual herbaceous^5^. In 2015 and 2016, seventy-five surface soil samples at 15 sites (5 samples per site) were collected for further wind tunnel experiments, particle size distribution analysis, and nutrient level analysis. More details of the regional environments and sampling strategies are provided in S1 and S2.

Wind tunnel experiments were conducted in the Key Laboratory of Desert and Desertification, Chinese Academy of Sciences, China. During the wind tunnel experiments, the samples were air-dried and the relative humidity was between 30 and 50%, similar to the values measured in the field at the sampling sites. More details of the wind tunnel experiments were described in S3. After all wind-tunnel experiments were completed, the aeolian materials collected were weighed using a balance with a precision of 0.001 g and were used for further particle size and nutrient level analyses. Particle-size distribution was measured using a Mastersizer 2000 (Malvern Co. Ltd., Malvern, UK; the sample range was between 0.02 and 2000 μm in diameter). Nutrient level analyses included measurements of the ammonium N, nitrate N, available K and available P, and the measurement methods are described in previous report^26^ and in S4.

Additionally, wind data from 1951 to 2015 at 15 stations located in the Mu Us Desert (Fig. 1) were used for further analyses (S5). These data were recorded in accordance with the World Meteorological Organization (WMO) and China National Meteorological Center (CNMC) standards. Because most datasets were complete after 1970, wind data records from 1971 to 2015 were used to evaluate the temporal variation in the aeolian transport potentials. More detailed descriptions of the methods are provided in S5.

## Electronic supplementary material

Supplementary Information
