# Supplementary material for: Key driving forces of desertification in the Mu Us Desert, China
Source: Sci Rep. 2017 Jun 21;7:3933. doi: 10.1038/s41598-017-04363-8 (PMC5479821; doi:10.1038/s41598-017-04363-8)
Supplement: Supplementary file 1 — Supplementary Information [file 41598_2017_4363_MOESM1_ESM.doc]

**Key driving forces of desertification in the Mu Us Desert, China**

*Xunming Wang^1, 2^, Hong Cheng^3^, Hui Li^4^, Junpeng Lou^1^, Ting Hua ^4^, Wenbin Liu^1^, Linlin Jiao^1^, Wenyong Ma^1^, Danfeng Li^1^, Bingqi Zhu^1^

^1^ Key Laboratory of Water Cycle & Related Land Surface Processes, Institute of Geographic Sciences and Natural Resources Research, Chinese Academy of Sciences, Beijing 100101, China

^2^ University of Chinese Academy of Sciences, Beijing 100049, China

^3^ State Key Laboratory of Earth Surface Processes and Resource Ecology, Beijing Normal University, Beijing 100875, China

^4^ Key Laboratory of Desert and Desertification, Cold and Arid Regions Environmental and Engineering Research Institute, Chinese Academy of Sciences, Lanzhou 730000, China

*Corresponding author: xunming@igsnrr.ac.cn

**Supplemental materials**

**S1 Desertification in the Mu Us Desert**

The Mu Us Desert of Central China (Figures.1 and S1) covers an area of 38,940 km^2^ ^21^ (or 91,700 km^2^ ^23^) in the UNEP map and is identified as a region of intense desertification and is affected by agricultural activities^1^. This desert is often characterized as being human-made over the past 2,000 years^27-32^. Researchers^32-34^ have attributed land degradation and vegetation rehabilitation to human activity. It is considered that the modern Mu Us Desert did not result principally from environmental evolution caused by climate forcing, but from intensive land use^35^. However, the “man-made” hypothesis has been challenged by the historical records of ancient China and archaeological evidence^36-38^; there have been at least 27 cycles of alternating depositions of aeolian dune sands and fluvio-lacustrine facies and/or paleosols over the last 150,000 years^39, 40^. Moreover, the aeolian sequences in this region were deposited over approximately 580,000 years^41, 42^, which suggests that this desert was not “human-made”. However, the key driving forces of desertification in the region still subject to argument.


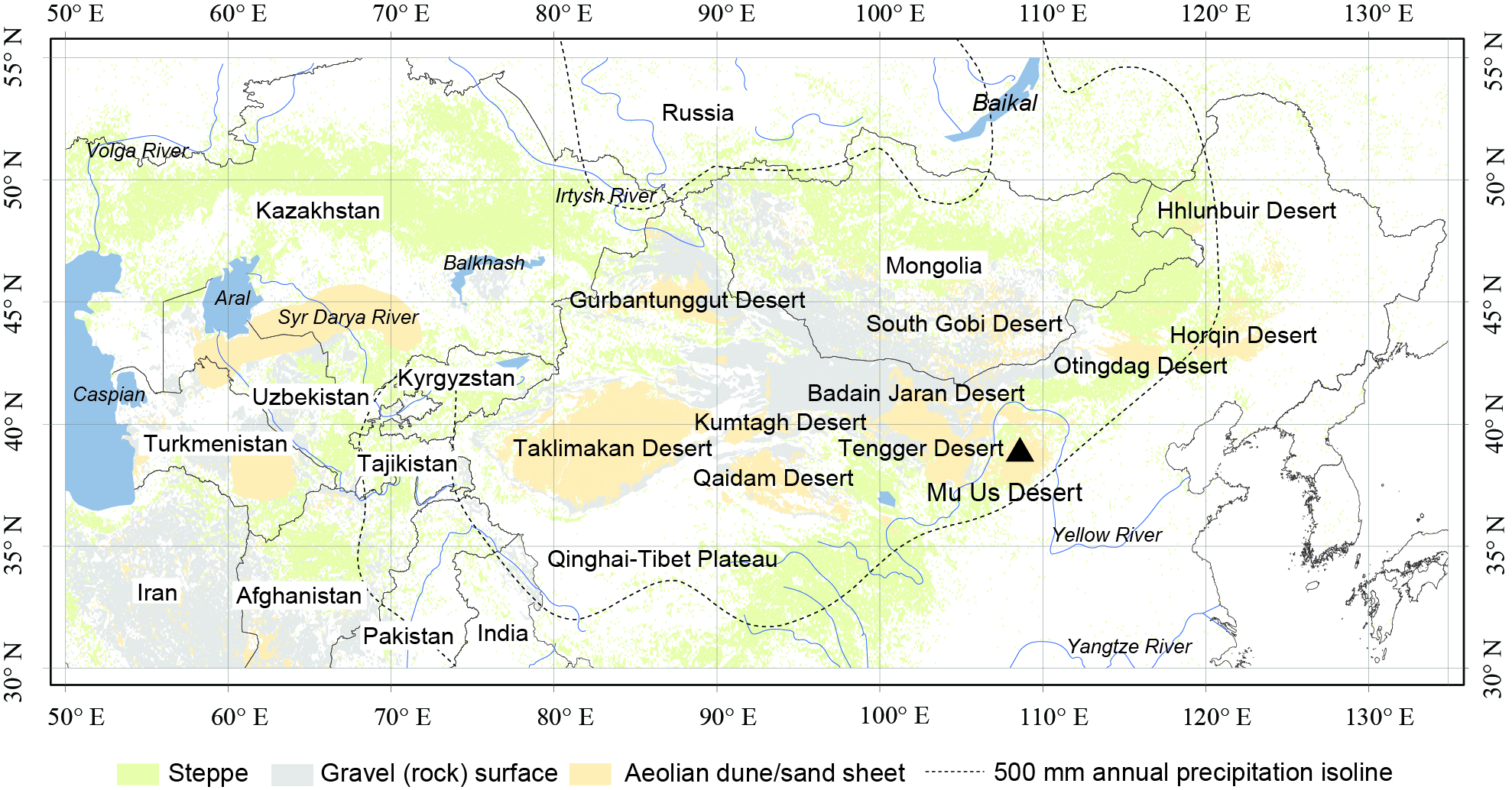


Figure S1. Location of the Mu Us Desert and the scopes of arid Asia (The figure was finished using Arcgis software (version 10.1, ESRI Inc., Redlands, California, USA), which can be downloaded from the internal network of Institute of Geographic Sciences and Natural Resources Research, Chinese Academy of Sciences.)

The plant species in this region mainly include *Artemisia ordosica, Sabina vulgaris, Salix mongolia, Salix cheilophyla, Hippophae rhamnoides*^28^ and large-scale cultivated crops. The desertification forms mainly include anchored or semi-anchored dunes/sand sheets reworked into mobile or semi-anchored dunes/sand sheets, and the arable land is covered by mobile sands (Table S1).

Table S1 Major desertification forms in the Mu Us Desert^23, 28^.

Note: The vegetation cover of anchored, semi-anchored, and mobile dunes are >40%, 15~40%, and <15%, respectively^28^.

| **Grade** | **Surface categories** | | |
| --- | --- | --- | --- |
|  | **Anchored or semi-anchored dune or sand sheet** | **Shrub** | **Farmland** |
| Grade 1 | Blowouts occur on the stoss slope of dunes; the total area of mobile sands is 5% to 20%, dunes anchored or semi-anchored | Under shrubs little mobile sand accumulates | Some sands accumulate in furrows, and erosion signals appear on plowing ridges |
| (Slight) |  |  |  |
| Grade 2 | Stoss and slip slopes appear, the total area of mobile sands is 20% to 50%, dunes semi-anchored | Mobile sands appear on the stoss side of mounds, some gravels and sand appear on inter-mounds | Patches of mobile sands appear, loss of thickness of humus layer is beyond 50% |
| (Moderate) |  |  |  |
| Grade 3 | Total area of mobile sands >50%, dunes semi-anchored | Vegetation cover <20% while area of mobile sands <50% | Humus layers are entirely lost, total area of mobile sand >20% and farmland is abandoned |
| (Severe) |  |  |  |
| Grade 4 | Mobile dunes or sand sheets, vegetation cover <5%, dunes shifted | Mobile dunes or sand sheets appear with vegetation cover <5% | Mobile sand sheets or gravel surface with vegetation cover <5% |
| (Very severe) |  |  |  |

Although there have been some disagreements about desertification trends, there has been obvious temporal variation in desertification in the Mu Us Desert over recent decades. For example, some studies^23^ have shown that there were no obvious variations in the total areas of desertification in the Mu Us Desert from the mid-1970s to 2010. The substantial decreases of areas affected by grade 4 desertification (Table S2) and monitoring data^15, 16^ suggest that extensive rehabilitation has occurred recently. Additionally, among the desertification grades^23^, only grade 4 classification may have played an important role in desertification and nutrient loss in the region. More details are shown in *S7*.

Table S2 Areas of desertification (km^2^) in the Mu Us Desert over recent decades^23^; the classification standards of desertification are shown in Table S1.

| Year | Grade 1 (Slight) | Grade 2 (Moderate) | Grade 3 (Severe) | Grade 4 (Very severe) |
| --- | --- | --- | --- | --- |
| Mid-1970s | 16234 | 11807 | 8401 | 13168 |
| 1990 | 14878 | 14165 | 12423 | 14260 |
| 2000 | 15867 | 12932 | 12045 | 16735 |
| 2005 | 16788 | 14507 | 13235 | 12403 |
| 2010 | 17044 | 15138 | 15002 | 9235 |

**Note:** Although study^23^ showed that from the mid-1970s to 2010 grade 1-3 desertification increased, it has been reported that from 2000 to 2015 the areas of desertification with those grades decreased around and in the Mu Us Desert region^15, 16, 43, 44^.

**S2 Field sampling criteria**

We collected surface materials from the Mu Us Desert during 2015 and 2016 (Figure 1). At each site, 5 representative samples were collected involving different vegetation cover and land use types (steppes, shrubs, and farmlands). A total of 75 samples in 15 sites were used for further wind tunnel experiments. The other sampling criteria including the geomorphologic characteristics of sampling sites are typical landscapes within at least a 10×10 km^2^ area. At each site, the samples were collected at horizontal intervals ranging from 100 to 200 m. The landscapes of the sampling sites are shown in Figure S2.


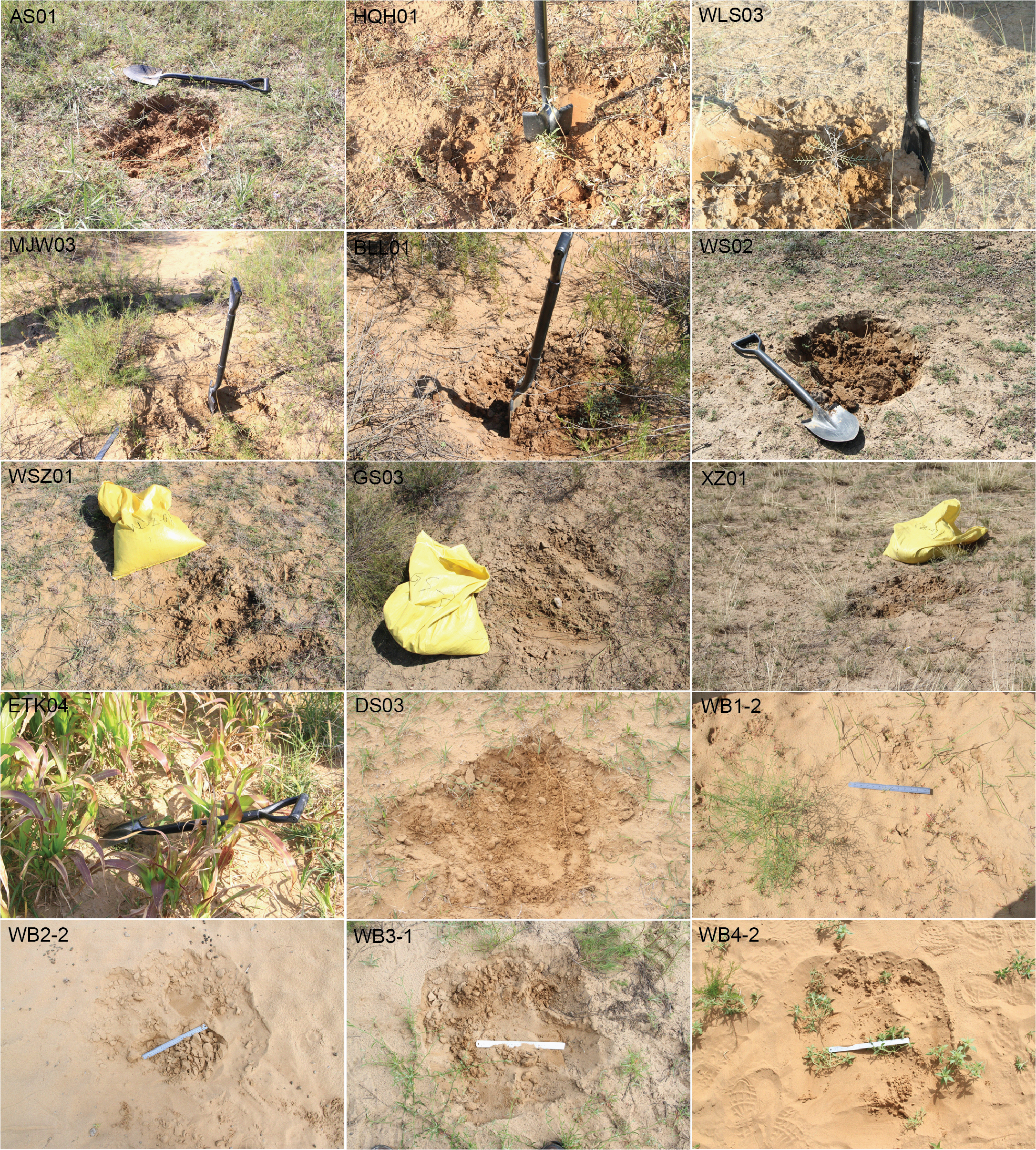


Figure S2. Landscapes of each sampling site

**S3 Wind tunnel experiments**

Wind tunnel experiments were carried out at the Key Laboratory of Desert and Desertification of the Cold and Arid Regions Environmental and Engineering Research Institute, Chinese Academy of Sciences, China. The size and operating characteristics of the wind tunnel have been described in detail by previous reports^45, 46^; the wind tunnel experiment processes and the sample layout are shown in Figure S3. For each sample, we positioned the sample in the working section of the wind tunnel with sizes of 150×30 cm, with the surface at the same level as the bottom of the wind tunnel. At 30 cm downwind from the sample, we installed a sand trap that was 30 cm wide (i.e., the same width as the surface samples) by 30 cm tall to collect windblown materials. Experiments began with a free-stream wind velocity of 8 m s^-1^, and the above processes were then repeated by increasing wind velocity at increments of 2 m·s^-1^ until the wind velocity reached 22 m·s^-1^.


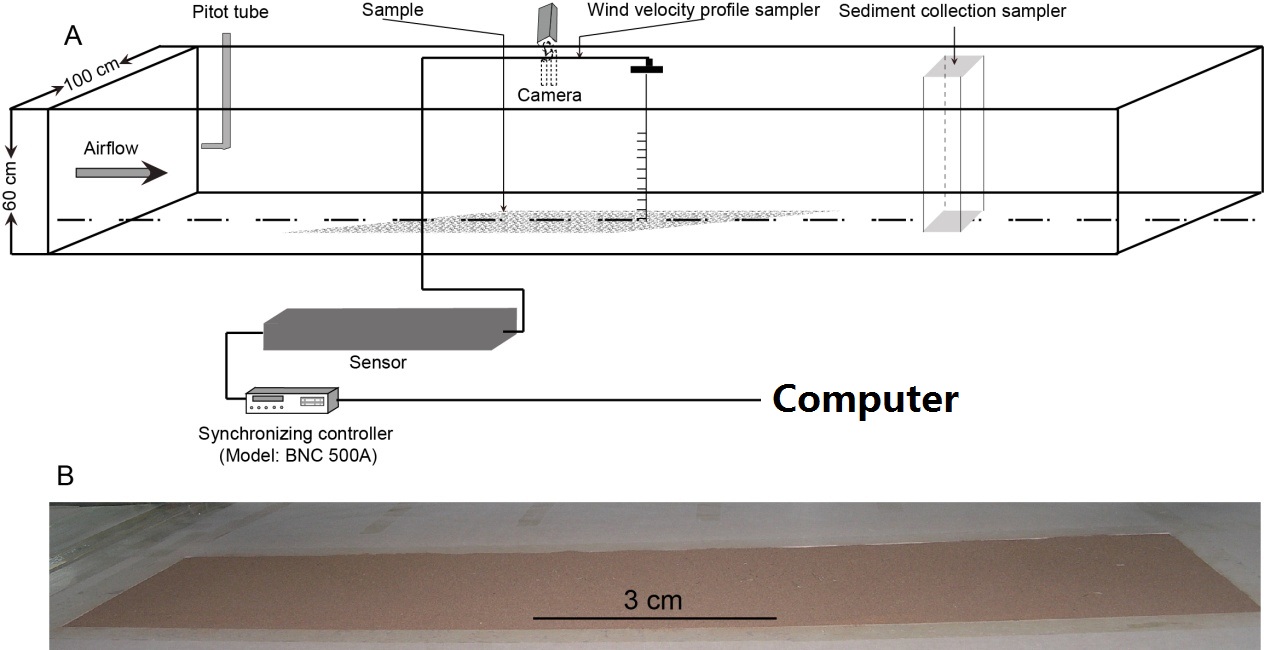


Figure S3. Schematic diagram of the wind tunnel (A) and sample arrangement (B) in the wind tunnel experiments.

**S4 Sample analyses**

After the wind tunnel experiments were completed, transported sediments collected by the sediment trap were weighed using a high-precision balance for further analyses of particle-size and nutrient levels. A Mastersizer 2000 instrument (Malvern Co. Ltd., Malvern, UK; the sample range was between 0.02 and 2,000 μm in diameter) was used to measure the particle size distribution. Previous report^47^ provided the comparisons of the measured results using this method with the results of other methods such as sieving, hydrometer, and pipette methods. Before the particle size measurements, we immersed the sediments in 10% H_2_O_2_ followed by immersion in 12.7% HCl to remove any plant debris and to disperse aggregates within the sediments, and the sample residue was treated with 10 mL of 0.05 M (NaPO_3_)_6_ on an ultrasonic vibrator for 10 min to facilitate dispersion. Nutrients, including ammonium N, nitrate N, available K, and available P, were measured using the common methods^26^. Briefly, during measurements all soil samples were air-dried, ground and passed through a 1-mm sieve. Soil nitrate and ammonium concentrations were extracted with 2 M KCl, and were determined with a UV-vis spectrophotometer (HACH DR5000, USA). Soil available P and available K were extracted with ammonium carbonate and ammonium carbonate, and were measured with ICP-OES (Optima 5300DV, USA).

**S5 Data processing**

The wind data based on the World Meteorological Organization (WMO) standards for anemometer heights (10 to 12 m) at 02:00, 08:00, 14:00, and 20:00+08:00 GMT from 15 meteorological stations located in the Mu Us Desert (Figure 1) were used for further analyses. Because most datasets were complete after 1970, we used data records from 1971 to 2015 to determine the temporal variation in the aeolian transport potential. According to our previous studies^5, 48, 49^, the threshold velocity in most Chinese sand desert regions is 6 m s^-1^. Therefore, only the frequency of wind velocity exceeding the threshold velocity was used in further discussion. To have enough grades to carry out wind tunnel experiments, the wind data were counted into groups of 5~7, 7~9, 9~11, 11~13, 13~15, 15~17, 17~19, and 20 m s^-1^, and the corresponding wind velocities in wind tunnel experiments were 6, 8, 10, 12, 14, 16, 18, and 20 m s^-1^. Therefore, the experimental wind velocities in the wind tunnel were set as 8, 10, 12, 14, 16, 18, and 20 m s^-1^, respectively. Although there were some differences between aeolian transport rates from wind tunnel experiments and the field observations under the same wind velocity, there were no significant differences in their variation tendencies with wind velocity^50, 51^. However, in the field and in natural conditions, there may have been lower contents of loose and fine particles on intact surfaces than on disturbed samples because of the bonding abilities of particles, the cover of coarse fractions on the fine particles, and the differences in airflow structures. Therefore, aeolian transport rates in wind tunnel experiments may be higher than in the field. Additionally, the correlation analyses showed that there were no significant correlations between the different particle fractions and the contents of ammonium N, nitrate N, available K, and available P (Table S3) (except for some fractions in available P and available K), which suggests that the contents of nutrients in the transported materials could be acquired from its contents in surface samples and the amounts of the transported materials.

Table S3 Correlations between the nutrient components and the different particle size fractions of surface soils.

| **Correlations** | | | | |
| --- | --- | --- | --- | --- |
|  | Ammonium N | Nitrate N | Available K | Available P |
| Mean particle size | -.098 | .245 | -.275^*^ | -.030 |
| < 2.5 μm | .112 | -.034 | .231 | .120 |
| < 5 μm | .097 | -.035 | .193 | .104 |
| < 10 μm | .087 | -.036 | .175 | .100 |
| < 50 μm | .116 | -.029 | .273^*^ | .176 |
| 50~100 μm | .060 | -.002 | .533^**^ | .279^*^ |
| 100~200 μm | -.029 | -.205 | .163 | -.054 |
| 200~250 μm | -.080 | -.211 | -.374^**^ | -.336^*^ |
| >250 μm | -.063 | .194 | -.382^**^ | -.105 |

*Correlation is significant at the 0.05 level (2-tailed).

**Correlation is significant at the 0.01 level (2-tailed).

The in situ nutrient loss of surface soils occurred under aeolian processes after the surface soils were eroded, transported, and deposited in far away from the source regions. After the wind tunnel experiments, sample analyses, and wind data processing were completed, the raw data acquired during the experiments and the statistics were re-processed. The nutrient losses in different wind velocities were summed to determine the total nutrient loss per unit, and finally, the key roles of desertification from the 1970s to 2015 were analyzed.

**S6 Determination of the nutrient loss triggered by desertification**

In China, dunes with vegetation cover below 15% are classified as mobile^28^. However, the vegetation cover thresholds for aeolian transport to occur on dune and sand sheet surfaces were usually above 14%^52-55^. Over the past several decades, although desertification with different grades occurred in the Mu Us Desert, grade 1-3 desertification processes may have made no significant contribution to the nutrient loss because at those stages the vegetation cover was far higher than 14%. Therefore, although desertification may have occurred on the surfaces, only areas with grade 4 desertification resulted in high nutrient loss.

Although there were no significant variations in the total areas of desertification in the Mu Us Desert from the mid-1970s to 2010, high levels of rehabilitation still occurred in the region. Additionally, the areas of grade 4 desertification decreased substantially after 2005, resulting in a dramatic decline in nutrient loss. Considering the desertification trends over the past several decades^23^, we propose that the degree of desertification in the mid-1970s, 1990, 2000, 2005, and 2010 represents the aeolian transport potentials in the 1970s, 1980s, 1990s, 2000s, and 2011~2015, respectively.

**References:**

1. Hou, R. Mu Us Desert evolution as indicated by the deserted ancient cities along the Sjara River. *Cultural Relic* ***1****,* 35–41 (1973).
2. Zhu, Z., Wu, Z., Liu, S. & Di, X. An induction to Chinese deserts. Science Press, Beijing. 107 pp (1980).
3. Zhu, Z. & Liu, S. Desert evolutions in historical periods in China. In: Chinese Natural Geography (Vol. Historical Geography) (Eds. by Zhu, Z. and Liu, S.), 249–260. Science Press: Beijing (1982).
4. Wang, S. Historical changes of pastoral and farming economy in Ordos Plateau and its impacts on natural environment. *Historical Geography* **5**, 11–24 (1985).
5. Jing, A. An introduction to desert archaeology. Forbidden City Publishing House: Beijing, 136–176 (2000).
6. Wu, W. Study on processes of desertification in Mu Us Sandy Land for last 50 years, China. *Journal of Desert Research* **21**, 164–169 (2001).
7. Wu, W., Wang, X. & Yao, F. Applying remote sensing data for desertification monitoring in the Mu Us Land. *Journal of Desert Research* **17**, 415–420 (1997).
8. Wu, B. & Ci, L. Landscape change and desertification development in the Mu Us Sandland, Northern China. *Journal of Arid Environments* **50**, 429–444 (2002).
9. Sun, J., Ding, Z. & Yuan, B. Desertification of Mu Us region since 2000a B. P. *Arid Land Geography* **18**, 36–42 (1995).
10. Zhao, Y. Mu Us Desert evolution in historical periods. *Historical Geography* **1**, 34–47 (1981).
11. Wang, W. When Maowusu became a desert? –view through new archaeological finds. *Archaeology and Cultural Relic* **5**, 80–85 (2002).
12. Han, Z. The evolution of the Mao Wusu Desert and the reclamation in the adjacent areas in the Ming Dynasty. *China Social Sciences* **5**, 191–204 (2003).
13. Dong, G., Gao, S., Jin, J. & Li, B. Formation, evolution, and origin of Mu Us Desert. *Science in China* **6**, 633–642 (1988).
14. Li, B. *et al*. Paleo-monsoon activities of Mu Us Desert, China since 150 ka B.P.— a study of the stratigraphic sequences of the Milanggouwan Section, Salawusu River area. Palaeogeography, Palaeoclimatology, Palaeoecology **162**, 1–16 (2000).
15. Sun, J., Yin, G., Ding, Z., Liu, T. & Chen, J. Thermoluminescence chronology of sand profiles in the Mu Us Desert, China. *Palaeography, Palaeoclimatology, Palaeoecology* **144**, 225–233 (1998).
16. Sun, J., Ding, Z., Liu, T., Rokosh, D. & Rutter, N. 580,000-year environmental reconstruction from aeolian deposits at the Mu Us Desert margin, China. *Quaternary Science Reviews* **18**, 1351–1364 (1999).
17. SFAC. http://wenku.baidu.com/link?url=pSeAi7Tw9Zl4wn-Nnv9iM6SaHGn_-6PABax0CYSiPNxCbgUAv XE0dyUJMkF2NtWI2VGCoPxoS-IIH83wgfNvfZKGFcowNyIvHf_J0LjqI0q (2000)
18. SFAC. <http://max.book118.com/html/2016/0114/33245246.shtm> (2005)
19. Dong, Z., Wang, H., Liu, X. & Wang, X. The blown sand flux over a sandy surface: a wind tunnel investigation on the fetch effect. *Geomorphology* **57**, 117–127 (2004).
20. Wang, X., Wang, G., Lang, L., Hua, T. & Wang, H. Aeolian transport and sandy desertification in semiarid China: A wind tunnel approach. *Land Degradation & Development,* doi: 10.1002/ldr.2249 (2013).
21. Eshel, G., Levy, G. J., Mingelgrin, U. & Singer, M. J. Critical evaluation of the use of laser diffraction for particle-size distribution analysis. *Soil Science Society of America Journal* **68**, 736–743 (2004).
22. Wang, X., Dong, Z., Yan, P., Zhang, J. & Qian, G. Wind energy environments and dunefield activity in the Chinese deserts. *Geomorphology* **65**, 33–48 (2005).
23. Wang, X., Hasi, E., Zhou, Z. & Liu, X. Significance of variations in the wind energy environment over the past 50 years with respect to dune activity and desertification in arid and semiarid northern China. *Geomorphology* **86**, 252–266 (2007).
24. Bagnold, R. A. The physics of blown sand and desert dune. Methuen, London (1941).
25. Sherman D.J. *et al*. Recalibrating aeolian sand transport models. *Earth Surface Processes and Landforms* **38**, 169-178 (2013).
26. Wasson, R. J. & Nanninga, P. M. Estimating wind transport of sand on vegetation surface. *Earth Surface Processes and Landforms* **11**, 505–514 (1986).
27. Wolfe, S. A. & Nickling, W. G. The protective role of sparse vegetation in wind erosion. *Progress in Physical* *Geography* **17**, 50–68 (1993).
28. Wiggs, G. F. S., Thomas, D. S. G., Bullard, J. E. & Livingstone, I. Dune mobility and vegetation cover in the southwest Kalahari Desert. *Earth Surface Processes and Landforms* **20**, 515–529 (1995).
29. Wiggs, G. F. S., Livingstone, I., Thomas, D. S. G. & Bullard, J. E. Airflow and roughness characteristics over partially vegetated linear dunes in the southwest Kalahari Desert. *Earth Surface Processes and Landforms* **21**, 19–34 (1996).
